# Pneumococcal serotypes and risk factors in adult community-acquired pneumonia 2018–20; a multicentre UK cohort study

**DOI:** 10.1016/j.lanepe.2023.100812

**Published:** 2023-12-11

**Authors:** Louise Lansbury, Hannah Lawrence, Tricia M. McKeever, Neil French, Stephen Aston, Adam T. Hill, Harry Pick, Vadsala Baskaran, Rochelle C. Edwards-Pritchard, Lesley Bendall, Deborah Ashton, Jo Butler, Priya Daniel, Thomas Bewick, Chamira Rodrigo, David Litt, Seyi Eletu, Carmen L. Sheppard, Norman K. Fry, Shamez Ladhani, Caroline Trotter, Wei Shen Lim

**Affiliations:** a Faculty of Medicine and Health Sciences, University of Nottingham, Nottingham, UK; b National Institute for Health Research (NIHR) Nottingham Biomedical Research Centre, UK; c Liverpool University Hospitals NHS Foundation Trust, Liverpool, UK; d Institute of Infection Veterinary & Ecological Science, University of Liverpool, UK; e Centre for Inflammation Research, University of Edinburgh, UK; f Respiratory Medicine, University Hospitals of Derby and Burton NHS Foundation Trust, Derby, UK; g Department of Respiratory Medicine, Nottingham University Hospitals NHS Trust, Nottingham, UK; h Respiratory and Vaccine Preventable Bacteria Reference Unit, UK Health Security Agency, Colindale, UK; i Immunisation and Vaccine Preventable Diseases, UK Health Security Agency, Colindale, UK; j Disease Dynamics Unit, Department of Veterinary Medicine, University of Cambridge, Cambridge, UK

**Keywords:** Pneumonia, *Streptococcus pneumoniae*, Serogroup, Pneumococcal vaccines, Risk factors

## Abstract

**Background:**

Higher-valency pneumococcal vaccines are anticipated. We aimed to describe serotype distribution and risk factors for vaccine-serotype community-acquired pneumonia (CAP) in the two years pre-SARS-CoV-2 pandemic.

**Methods:**

We conducted a prospective cohort study of adults hospitalised with CAP at three UK sites between 2018 and 2020. Pneumococcal serotypes were identified using a 24-valent urinary-antigen assay and blood cultures. Risk factors associated with vaccine-type pneumonia caused by serotypes in the 13-, 15- and 20-valent pneumococcal conjugate vaccines (PCV13, PCV15, PCV20) and 23-valent pneumococcal polysaccharide vaccine (PPV23) were determined from multivariable analysis.

**Findings:**

Of 1921 adults hospitalised with CAP, 781 (40.7%, 95% confidence intervals (CI) 38.5–42.9%) had pneumococcal pneumonia. A single PCV13-serotype was detected in 242 (31.0%, 95% CI 27.8–34.3%) pneumococcal CAP patients, mostly serotype 3 (171/242, 70.7%, 95% CI 64.5–76.0%). The additional two PCV15-serotypes were detected in 31 patients (4%, 95% CI 2.8–5.6%), and PCV20-non13-serotypes in 192 (24.6%), with serotype 8 most prevalent (123/192, 64.1%, 95% CI 57.1–70.5%). Compared to PCV13-serotype CAP, people with PCV20-non13 CAP were younger (median age 62 versus 72 years, p < 0.001) and less likely to be male (44% versus 61%, p = 0.01). PPV23-non13-serotypes were found in 252 (32.3%, 95% CI 29.1–35.6%) pneumococcal CAP patients.

**Interpretation:**

Despite mature infant pneumococcal programmes, the burden of PCV13-serotype pneumonia remains high in older adults, mainly due to serotype 3. PCV20-non13-serotype pneumonia is more likely in younger people with fewer pneumococcal risk factors.

**Funding:**

Unrestricted investigator-initiated research grant from 10.13039/100004319Pfizer, United Kingdom; support from 10.13039/501100000272National Institute for Health Research (NIHR) Biomedical Research Centre, Nottingham.

**Research in context**

**Evidence before this study**

A published systematic review of vaccine serotypes in non-invasive pneumococcal pneumonia in older adults, searching PubMed, EMBASE and MEDLINE for studies published in any language between 1 January 1990 and 30 March 2021, using search terms relating to “community-acquired pneumonia” OR “*Streptococcus pneumoniae*” AND “pneumococcal serotypes” identified twenty-eight eligible studies from Europe, North America and Asia. During the one to five years following the introduction of childhood PCV10/13 immunisation, the pooled estimate of the proportion of adult pneumococcal pneumonia due to PCV13 vaccine serotypes was 49% (95% confidence interval 43%–54%), with estimates influenced by method of serotype detection and with higher estimates from Europe compared to North America. Extending the search in PubMED to identify studies published up to 1 September 2023 identified additional studies reporting coverage for PCV15 serotypes ranging from 34% to 45% and for PCV20 serotypes from 58% to 69%. Data on the risk factors associated with pneumonia caused by the additional serotypes in newer pneumococcal vaccines are important to the formulation of vaccine policy but are currently lacking.

**Added value of this study**

To our knowledge this is the largest ongoing study of pneumococcal serotype distribution in hospitalised community-acquired pneumonia patients in the UK, which uses a 24-valent serotype-specific urinary antigen detection assay for enhanced diagnosis of pneumococcal pneumonia. We found that in almost a third of patients with pneumococcal pneumonia, a single PCV13 serotype was implicated, 71% of which were serotype 3. The additional PCV15 serotypes were found in a further 4% of patients with pneumococcal pneumonia, and PCV20 serotypes not covered by PCV13 in a further 24.6% of patients, with serotype 8 accounting for 64% of these. Our study provides evidence on the burden of potentially vaccine-preventable pneumococcal CAP in adults.

**Implications of all the available evidence**

Pneumococcal pneumonia in adults remains a major reason for hospitalisation in the UK and a high proportion of pneumococcal pneumonia is due to serotypes potentially covered by novel higher-valency pneumococcal conjugate vaccines. Consideration should therefore be given to direct immunisation of adults with these new-generation vaccines.

## Introduction

Community-acquired pneumonia (CAP) is a major cause of morbidity and mortality worldwide, with *Streptococcus pneumoniae* (pneumococcus) being the commonest causative pathogen.^1^ Although pneumococcus also causes invasive diseases such as meningitis, sepsis and bacteraemic pneumonia, the greatest burden is in older adults with pneumococcal CAP, the majority of which are non-bacteraemic.^2^^,^^3^

One hundred pneumococcal serotypes have now been identified.^4^ Two types of serotype-specific pneumococcal vaccine are currently available to help protect against invasive and non-invasive disease; pneumococcal conjugate vaccines (PCVs) and a 23-valent pneumococcal polysaccharide vaccine (PPV23). In 2006, a seven-valent PCV (PCV7) was introduced to the United Kingdom (UK) childhood immunisation schedule and replaced with a 13-valent vaccine (PCV13) in 2010.^5^ Since 2003, the PPV23 vaccine has been recommended for adults aged ≥65 years and those aged ≥2 years in a clinical risk group for pneumococcal disease,^5^ with moderate protection against PPV23 serotype invasive pneumococcal disease (IPD)^6^ and pneumonia.^7^ PCV implementation has been associated with large declines in vaccine-serotype IPD across all age groups due to direct and indirect protection, but with an increase in disease caused by non-PCV serotypes (serotype replacement).^8^^,^^9^ A recent systematic review of vaccine serotypes in older adults reported that 18% of hospitalised CAP cases were attributable to pneumococcus, with a higher proportion in Europe (26%) compared to North America (11%), and that even after the introduction of PCV10/13 vaccines into national childhood immunisation programmes, almost half of pneumococcal CAP was caused by PCV13 serotypes.^10^

Since 2008, we have been conducting a prospective cohort study of pneumococcal pneumonia in adults in Nottingham, UK. We previously reported that the incidence of non-invasive pneumococcal pneumonia has been increasing, predominantly due to serotype 3 and non-PCV13 serotypes.^11^ With the recent licensure of next-generation higher-valent PCVs (PCV15, PCV20) for adults,^12^^,^^13^ it is important to evaluate the serotypes responsible for pneumococcal pneumonia and the risk factors associated with vaccine-serotype disease to better inform vaccine policy.

In this UK multicentre study, we describe pneumococcal serotype distribution in hospitalised adults with pneumococcal CAP in the 24-months prior to the SARS-CoV-2 pandemic, and evaluate the risk factors associated with pneumococcal pneumonia caused by serotypes included in the different pneumococcal vaccines.

## Methods

### Study design

A prospective multi-centre cohort study was conducted of consecutive adult patients admitted with community-acquired pneumonia (CAP) to four university hospitals at three sites in the UK, Nottingham (two hospitals), Liverpool and Edinburgh, between April 2018 and March 2020, building upon the previously-reported single centre study during 2008–18.^11^^,^^14^ Acute admissions were screened for study eligibility each weekday and reviewed within 48 h of admission by the study team. Eligibility for inclusion in the study was defined as patients aged ≥16 years presenting with ≥1 symptoms associated with a lower respiratory tract infection (cough, increasing dyspnoea, sputum production and/or fever), with acute abnormalities consistent with infection on a chest radiograph taken within 48 h of admission, and treated as CAP. Exclusion criteria included hospitalisation in the ten days prior to the index admission, and post-obstructive pneumonia secondary to lung cancer (See also Appendix 1, online supplement.).

### Microbiology

Blood cultures and microbiological investigation of respiratory samples were conducted at the discretion of the clinical team. Additional urine samples were obtained within 48 h of admission for pneumococcal-specific urinary antigen testing (Bio-Plex24 assay, see Appendix 1 in online supplement for details). The Binax-NOW® (Alere, Stockport, UK) assay for pneumococcal C-polysaccharide urinary antigen detection (UAD) was performed at the local microbiology laboratory at the Nottingham and Liverpool sites only.

Patients were considered to have pneumococcal CAP if they met ≥1criteria: a) positive pneumococcal UAD or b) blood culture positive for *S pneumoniae* or c) detection of a pneumococcal serotype or cell wall polysaccharide by the Bio-Plex24 assay.

### Statistical analysis

All consented patients who had radiologically-confirmed CAP were included in the analysis. Serotypes were classified according to the serotype content of pneumococcal vaccines: PCV13 serotypes (1, 3, 4, 5, 6A, 6B, 7F, 9V, 14, 18C, 19A, 19F, 23F), PCV15 serotypes (PCV13 serotypes plus 22F and 33F), PCV20non13 serotypes (8, 10A, 11A, 12F, 15B, 22F, 33F), PPV23non13 serotypes (2, 8, 9N, 10A, 11A, 12F, 15B, 17F, 20, 22F, 33F) and non-vaccine types (NVT) (any non-PCV13, non-PPV23 serotypes, or non-typeable disease in which cell-wall polysaccharide was detected but the Bio-Plex24 assay was not able to generate a serotype-specific result), or ‘untyped’ disease in which UAD was positive without subsequent serotype identification). Patients with multiple serotypes detected in the same sample were excluded from analyses of clinical risk groups and clinical characteristics if the serotypes detected crossed the vaccine classifications being assessed.

Baseline characteristics and comorbidities were compared for people with disease due to serotypes in the different vaccines with those whose serotypes were outside the corresponding vaccine. Pearson's χ2 or Fisher's exact tests were used to compare categorical variables, and the Mann–Whitney U-test for non-normally distributed continuous variables. Multivariable logistic regression was used to compare the independent association between comorbidity and a) PCV13 b) PCV15 c) PCV20-nonPCV13 serotype, and d) PPV23-non13 serotype CAP. Variables for inclusion in the multivariable analysis were purposefully selected based on clinical risk factors outlined in the UK Health Security Agency's “Immunisation against Infectious Disease” (The Green Book)^5^ and previous studies.^15^ We adjusted for age, sex, residential care status, previous vaccination with PPV23 (from primary care or pharmacy records), and comorbidities (malignancy, chronic liver disease, cerebrovascular disease, diabetes mellitus, chronic lung disease, chronic heart disease, immunosuppression and chronic kidney disease). Additionally, the risk of pneumococcal CAP with serotypes in the different vaccines was analysed for the following clinical risk groups:

a) specific clinical risk groups (i) aged 16–64 years with no clinical risk factors (Clinical risk factors include chronic respiratory disease, chronic heart disease, chronic liver disease, chronic kidney disease, diabetes requiring treatment, immunosuppression (immunosuppressive medications, previous solid organ transplant, previous bone marrow transplant, splenic dysfunction, neoplastic disease with active treatment in last 6 months, haematological malignancy, primary immunodeficiency, HIV), CSF leak or cochlear implant) (ii) aged 16–64 years with one or more clinical risk factor (iii) aged ≥65 years with no clinical risk factors (iv) aged ≥65 years with ≥1 clinical risk factors;
b) pneumococcal clinical risk group, defined as adults aged 16–64 years with ≥1 clinical risk factors or any adult aged ≥65 years (i.e. an amalgamation of clinical risk groups ii, iii, and iv above);
c) increasing number of clinical risk factors (none to ≥3, excluding age).

To investigate the impact of serotype 8 on the adjusted point estimates of PCV20-non13 versus non-PCV20-non13 serotype CAP, we conducted a sensitivity analysis in which patients with serotype 8 were excluded from the PCV20-non13 cohort. All the regression analyses used multiple imputation to account for missing data with five imputations and accounted for potential clustering by site by using the cluster option in logistic regression (See online Appendix 1 for more information on the multiple imputation model). We also ran a multilevel mixed-effects logistic regression model to examine for heterogeneity across the sites, but this did not make a difference to the overall results. Statistical analyses were conducted using Stata/SE V.17.0 (StataCorp. 2021).

### Ethics approval

The work was approved by the Nottingham Research Ethics Committee (REC reference 08/H0403/80).

### Role of the funding source

This study is independent research supported by the National Institute for Health Research Biomedical Research Centre and an unrestricted investigator-initiated research grant from Pfizer. Pfizer had no part in the design or execution of the study, analysis and interpretation of results, writing the manuscript or decision to submit for publication. The data are the sole responsibility of the authors and the study was sponsored by Nottingham University Hospitals NHS Trust.

## Results

### Study population

During the study period, 2249 potential participants were identified across the three sites (1605 Nottingham, 309 Liverpool, and 335 Edinburgh). Forty-two (1.9%) patients had an alternative diagnosis and consent was not obtained for 286 (12.7%) patients. People without consent were older (median age 82 years, interquartile range (IQR) 72–88 years versus 71 years, IQR 56–81 years), p ≤ 0.001), more often resident in care homes (32.5% versus 2.6%, p < 0.001), more likely to have co-morbidities, and have a higher CURB65 score (p for trend <0.001) compared with consented patients.

### Baseline characteristics

There were 1921 consented adults hospitalised with CAP between 16 April 2018 and 10 March 2020, with 150 patients (7.8%) admitted to a critical care unit (Table 1). The median age was 70.0 years (IQR 56.3–80.0 years), 1029 (53.6%) were male, and pneumonia severity based on the CURB65 score was low, moderate and high in 983 (51.2%), 581 (30.2%) and 357 patients (18.6%) respectively. At least one comorbidity was present in 1129 patients (58.8%). Overall 8.3% (159/1921) of the cohort were known to have died within 30 days. PPV23 vaccine receipt confirmed by primary-care or pharmacy records was recorded in 722/1287 patients (56.1%) aged 65 years or above or in a clinical risk group and with known vaccination status, with unknown vaccination status in eligible patients of 12.2% (178/1465). Median time since PPV23 vaccination was 10.9 years (IQR 4.0–14.9 years).

**Table 1:** Baseline characteristics, comorbid disease, severity, and outcomes of community-acquired pneumonia (CAP) and pneumococcal CAP cohort.

Patient characteristics	Whole cohort (n = 1921)	Pneumococcal cohort (n = 781)	Non-pneumococcal CAP (n = 1140)
**Demographics**			
Age, median; years (IQR)	70.0 (56.3–80.0)	69.7 (57.5–80.0)	71.0 (56.0–81.0)
16–49	314 (16.2)	117 (15.0)	197 (17.3)
50–64	437 (22.8)	196 (25.1)	241 (21.1)
65–74	449 (23.4)	188 (24.1)	261 (22.9)
75–84	441 (23.0)	176 (22.6)	265 (23.2)
≥85	279 (14.5)	103 (13.2)	176 (15.4)
Male (%)	1029 (53.6)	408 (52.2)	621 (54.5)
Residential care (%)	52 (2.7)	14 (1.8)	38 (3.3)
**Comorbid disease**			
Smoking status^a^			
Never smoked	561 (29.9)	210 (27.3)	351 (31.7)
Ex-smoker	932 (49.7)	375 (48.8)	557 (50.4)
Current smoker	382 (20.4)	184 (23.9)	198 (17.9)
Alcohol excess	44 (2.3)	11 (1.4)	19 (1.7)
COPD	553 (28.8)	258 (33.0)	295 (25.9)
Diabetes mellitus	338 (17.6)	139 (17.8)	199 (17.4)
Cerebrovascular disease	156 (8.1)	53 (6.8)	103 (9.0)
Chronic heart disease	396 (20.6)	151 (19.3)	245 (21.5)
Congestive cardiac failure	153 (8.0)	59 (7.6)	94 (8.2)
Ischaemic heart disease	311 (16.2)	116 (14.8)	195 (17.1)
Chronic lung disease (excluding asthma)	666 (34.5)	295 (37.8)	371 (32.5)
Asthma	241 (12.5)	108 (13.8)	133 (11,7)
Malignancy	154 (8.0)	59 (7.6)	95 (8.3)
Cognitive impairment	75 (3.9)	25 (3.2)	50 (4.4)
Liver disease	44 (2.3)	18 (2.3)	26 (2.3)
Kidney disease	210 (10.9)	87 (11.1)	123 (10.8)
Immunocompromised	85 (4.4)	38 (4.9)	41 (3.6)
Influenza vaccine in preceding 12 months	1205 (62.7)	492 (63.0)	713 (62.5)
PPV23 receipt	763 (39.7)	314 (40.2)	449 (39.4)
**Severity**			
CURB65 0–1 (low)	983 (51.2)	392 (50.2)	591 (51.8)
CURB65 2 (moderate)	581 (30.2)	223 (28.6)	358 (31.4)
CURB65 3–5 (high)	357 (18.6)	166 (21.2)	191 (16.8)
**Outcome**			
30-day mortality	159 (8.3)	59 (7.6)	100 (8.8)
30-day re-admission	275 (14.3)	122 (15.6)	153 (13.4)
LOS	6 (4–11)	6 (4–10)	6 (4–11)
ICU/HDU admission	150 (7.8)	64 (8.2)	86 (7.5)

Key: COPD Chronic obstructive pulmonary disease.

LOS Length of stay.

ICU/HDU Intensive care unit/high dependency unit.

CURB-65 score based on: confusion; urea ≥7 mmol/l; respiratory rate ≥30; systolic BP < 90 mmHg or diastolic BP ≤ 60 mmHg; age ≥65 years.

Data are presented as n (%) or median (IQR).

a Smoking status recorded for 1875 patients.

**Fig. 1:**
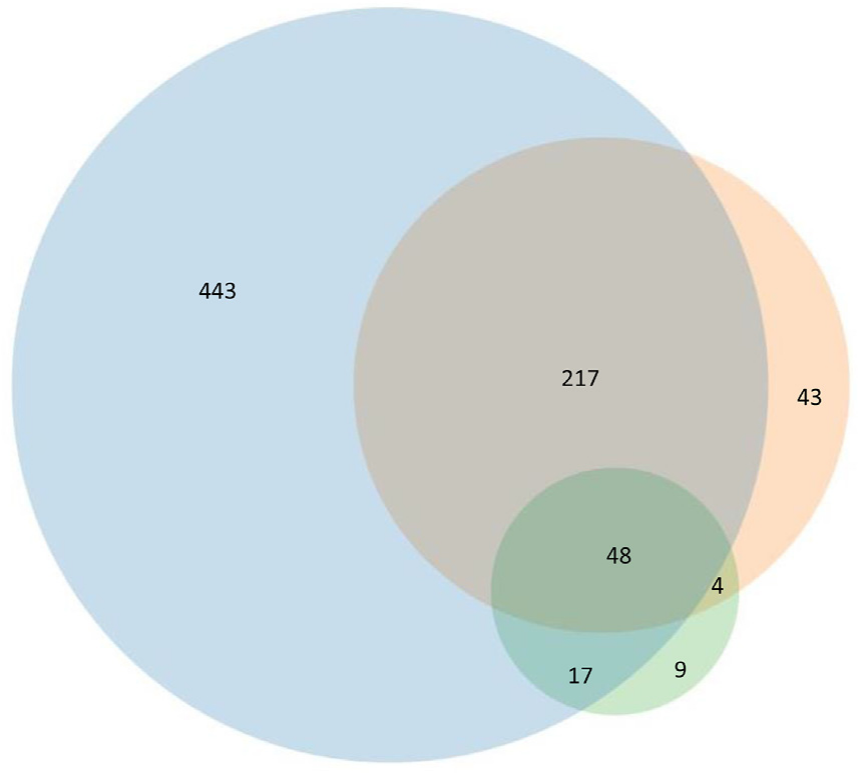
***Streptococcus pneumoniae* identification by diagnostic method among in 781 adults with pneumococcal community-acquired pneumonia. Circle areas are proportional to the number of positive samples by each detection method**. Key: Blue = Bio-Plex24; Orange = BinaxNow; Green = blood culture. Total number of people tested in the entire cohort: Bio-Plex24 n = 1694; BinaxNow n = 1380; Blood culture n = 1394.

Pneumococcal CAP was diagnosed in 781 (40.7%, 95% confidence intervals (CI) 38.5–42.2%) patients and included 78 (10.0%) with a positive pneumococcal blood culture. A Bio-Plex24 assay was performed in 1694/1921 (88.2%) of the entire cohort, and was positive in 725 of 760 (95.4%, 95% CI 93.4–96.7%) people with a diagnosis of pneumococcal pneumonia patients who were tested. Of 1380 patients who had a BinaxNOW test (Nottingham and Liverpool sites only), 649 had pneumococcal CAP and the BinaxNOW test was positive in 312 (48.1%) of these. *S pneumoniae* was detected by Bio-Plex24 alone in 56.7% (n = 443) of patients, BinaxNow alone in 5.5% (n = 43) and blood culture alone in 1.2% (n = 9) (Fig. 1). Other pathogens detected in the CAP cohort are described in Supplementary Figure S1.

### Pneumococcal serotypes

Details of pneumococcal serotyping results by test type are outlined in Appendix 2 of the online supplement.

Multiple serotypes were detected in 149/781 (19.1%) pneumococcal CAP patients. A single PCV13 serotype was identified in the Bio-Plex24 assay in 242/781 (31.0%, 95% CI 27.8–34.3%) patients with pneumococcal CAP; serotype 3 being most common (n = 171, 70.4% of PCV13 serotypes), followed by serotype 19A (n = 41, 16.9%). Multiple PCV13 serotypes which did not cross vaccine class were detected in 15 patients (1.9%).

A single PCV15 serotype was detected in 273/781 (35.0%, 95% CI 31.7–38.4%) pneumococcal CAP patients. The additional PCV15 serotypes (22F and 33F) were detected alone in 4.0% (n = 31, 95% CI 2.8–5.6%) of the pneumococcal cohort, and with other serotypes in 1.8% (n = 14, 95% CI 1.1–3.0%).

A single PCV20 serotype was detected in 434 (55.6%, 95% CI 52.1–59.0%) of pneumococcal CAP. Serotypes in PCV20 but not in PCV13 were identified as a single serotype in 192 patients (24.6% of pneumococcal CAP cohort), serotype 8 representing more than two-thirds (n = 123, 64.1%), followed by serotype 11A (n = 20, 10.4%). A PCV20-non13 serotype plus one or more non-PCV20-non13 serotypes was detected in 118 patients, with serotype 8 most prevalent (71/118, 60.2%).

**Fig. 2:**
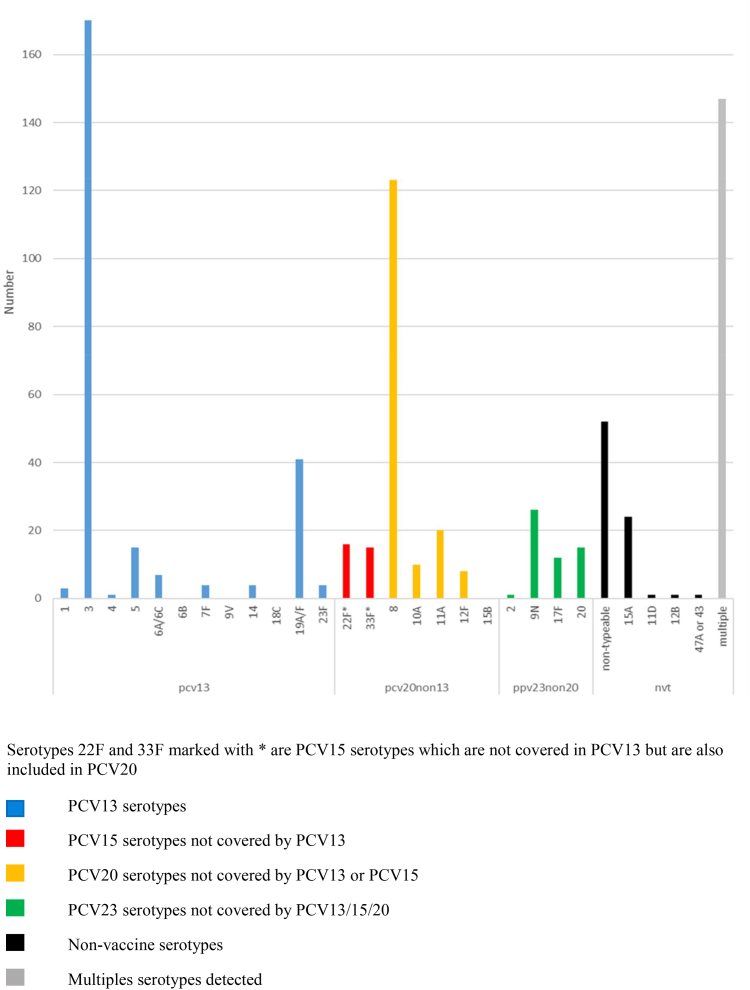
**Pneumococcal serotypes detected in patients by Bioplex-24 assay, grouped according to pneumococcal vaccine class in adults with pneumococcal CAP (N = 721). Non-typeable refers to the detection of pneumococcal cell-wall polysaccharide by the Bioplex-24 assay but an inability to define a particular serotype**. Serotypes 22F and 33F marked with ∗ are PCV15 serotypes which are not covered in PCV13 but are also included in PCV20.

Fifty-three patients (6.8% of pneumococcal CAP cohort) had a unique PPV23 serotype (not a PCV13 nor PCV20 serotype); 49.1% were serotype 9N, followed by serotype 20 (26.4%), and serotype 17F (22.6%). A NVT-serotype was detected in 86 patients (11.0%); 52 (60.5%) a non-typeable serotype, and 27.9% (n = 24) serotype 15A (Fig. 2). In 14/781 (1.8%) patients with CAP, more than two serotypes were detected but blood culture and BinaxNOW were both negative or not performed.

### PCV13-serotype pneumonia

Of 587 patients eligible for analysis (single serotype or multiple serotypes not crossing vaccine serogroup classes), 257 (43.8%, 95% CI 39.8–47.8%) had PCV13 serotype disease and 330 (56.2%) had non-PCV13 serotype disease. Patients with PCV13 serotype CAP were older than patients with non-PCV13 serotype CAP (median age 71.7 years, IQR 60.0–81.0 years versus 66.0 years, IQR 54.0–75.0 years, p = 0.002). The association with age persisted in people aged ≥65 years with no clinical risk factors (aOR 1.83, 95% CI 1.04–3.24, p = 0.04, n = 79, 13.4% of the included cohort). Compared to patients with non-PCV13 CAP, those with PCV13-serotype CAP were more likely to die within 30 days (aOR 1.70 (1.36–2.13, p < 0.001)) (Table 2). Male sex was also independently associated with PCV13 serotype CAP compared to non-PCV13 serotype CAP (aOR 2.11, 95% CI 1.89–2.35, p < 0.001) (Table 2).

**Table 2:** Clinical features of adults with single PCV13-serotype/multiple PCV13 serotypes compared to non-PCV13 serotype CAP amongst patients with pneumococcal pneumonia.

	Serotypes in PCV13 (n = 257)	Non–PCV13 serotypes (n = 330)	OR (95% CI)	p–value	aOR^b^ (95% CI)	p–value
Baseline characteristics						
Age^a^	71.7 (60.0–81.0)	66.0 (54.0–75.0)		**0.0002**		
Sex, male	157 (61.0)	148 (45.2)	1.98 (1.42–2.77)	**<0.001**	2.11 (1.89–2.35)	**<0.001**
Residential care	6 (2.3)	4 (1.2)	2.61 (0/74–9.19)	0.13	2.00 (0.59–6.79)	0.26
PPV23 vaccine receipt	113/228 (49.6)	117/302 (38.7)	1.56 (1.06–2.28)	**0.02**	1.65 (1.24–2.20)	**0.004**
Smoking						
Never	65/253 (25.7)	81/324 (25.0)	Ref	0.11^d^	Ref	0.48
Ex	137/253 (54.2)	149/324 (46.0)	1.14 (0.78–1.71)		1.12 (0.66–1.90)	
Current	51/253 (20.1)	94/324 (38.7)	0.68 (0.42–1.08)		1.16 (0.63–2.12)	
Malignancy	17 (6.6)	24 (7.3)	0.94 (0.44–2.04)	0.88	0.98 (0.44–2.19)	0.95
Liver disease	4 (1.5)	8 (2.5)	0.64 (0.19–2.14)	0.47	0.73 (0.35–1.51)	0.40
Chronic kidney disease	37 (14.4)	30 (9.1)	1.67 (1.00–2.79)	**0.05**	1.41 (0.80–2.47)	0.23
Chronic heart disease	54 (21.0)	63 (19.1)	1.13 (0.75–1.69)	0.56	0.88 (0.72–1.10)	0.26
Chronic cardiac failure	23 (8.9)	23 (7.0)	1.32 (0.72–2.42)	0.36	1.06 (0.60–1.88)	0.85
Ischaemic heart disease	38 (14.8)	52 (15.8)	0.91 (0.58–1.43)	0.67	0.45 (0.26–0.76)	**0.003**
Chronic lung disease	89 (34.6)	130 (39.4)	0.81 (0.58–1.14)	0.23	0.64 (0.52–0.80)	<**0.001**
COPD	78 (30.4)	114 (34.5)	0.82 (0.58–1.17)	0.28	1.06 (0.53–2.11)	0.87
Asthma	36 (14.0)	50 (15.2)	0.91 (0.57–1.45)	0.70	0.99 (0.73–1.34)	0.96
Diabetes	52 (20.2)	53 (16.1)	1.33 (0.87–2.03)	0.19	1.32 (0.73–2.41)	0.35
Cerebrovascular disease	18 (7.0)	19 (5.8)	1.23 (0.63–2.40)	0.54	0.85 (0.37–1.96)	0.70
Cognitive impairment	9 (3.5)	9 (2.7)	1.22 (0.62–2.38)	0.56	0.92 (0.71–1.21	0.56
Immunosuppression	17 (6.6)	16 (4.8)	1.39 (0.69–2.81)	0.36	1.04 (0.77–1.41)	0.79
Number of clinical risk factors						
0	94 (36.5)	132 (40.0)	Ref	0.37^d^	Ref	0.80^d^
1	96 (37.4)	119 (36.1)	1.13 (0.78–1.65)		0.96 (0.63–1.46)^e^	
2	49 (19.1)	60 (18.2)	1.15 (0.72–1.82)		0.91 (0.54–1.52)^e^	
≥3	18 (7.0)	19 (5.8)	1.33 (0.66–2.67)		1.05 (0.47–2.33)^e^	
Disease severity						
CURB score						
0–1 (low)	115 (44.7)	184 (55.8)	Ref	**0.002**^d^	Ref	0.88^d^
2 (moderate)	78 (30.4)	93 (28.2)	1.34 (0.91–1.96)		0.99 (0.60–1.64)	
≥3 (high)	64 (24.9)	53 (16.1)	1.93 (1.25–2.98)		1.45 (0.81–2.58)	
Critical care admission	21 (8.2)	27 (8.2)	1.00 (0.55–1.81)	0.99	1.01 (0.61–1.69)	0.97
30–day mortality	25 (9.7)	18 (5.4)	1.88 (1.00–3.54)	**0.047**	1.70 (1.36–2.13)	**<0.001**
30–day readmission	44 (17.1)	40 (12.1)	1.56 (0.97–2.50	0.06	1.57 (0.90–2.75)	0.11
Subgroup analyses for specific risk groups						
16–64 years, no clinical risk factor	47 (18.3)	97 (29.4)	0.54 (0.36–0.80)	**0.002**	0.67 (0.47–0.94)^e^	**0.02**
16–64 years, ≥1 risk factor	40 (15.6)	61 (18.5)	0.81 (0.52–1.26)	0.35	0.92 (0.49–1.75)^e^	0.81
≥65 years, no clinical risk factor	45 (17.5)	33 (10.0)	1.91 (1.18–3.10)	**0.009**	1.83 (1.04–3.24)^e^	**0.04**
≥65 years, ≥1 risk factor	124 (48.2)	139 (42.2)	1.28 (0.92–1.78)	0.14	0.99 (0.72–1.37)^e^	0.97
Pneumococcal clinical risk group^c^	209 (81.3)	233 (70.6)	1.15 (0.82–1.60)	0.43	0.94 (0.67–1.33)^e^	0.75

Key: OR Odds ratio; aOR adjusted odds ratio.

Data are presented as n (%). Bold values indicate p-value <0.05.

a Median age (interquartile range).

b Adjusted for age, sex, residential care, ppv23 vaccine receipt, comorbidities, number in adjusted model = 587.

c Pneumococcal clinical risk group includes everyone aged ≥65 years, plus anyone aged 16–64 years with one or more of the following clinical risk factors: chronic respiratory disease, chronic heart disease, chronic liver disease, chronic kidney disease, diabetes requiring treatment, immunosuppression (immunosuppressive medications, previous solid organ transplant, previous bone marrow transplant, splenic dysfunction, neoplastic disease with active treatment in last 6 months, haematological malignancy, primary immunodeficiency, HIV), CSF leak or cochlear implant.

d p-value for trend.

e Adjusted for sex, residential care, ppv23 vaccine receipt.

### PCV15 serotype pneumonia

Of 591 patients eligible for analysis, a PCV15 serotype was detected in 292 (49.4%, 95% CI 45.4–53.4%) patients. Serotypes 22F and 33F were detected as a single serotype in 16 (5.5%) and 15 (5.1%) patients with a PCV15 serotype respectively, and in a further 5 patients (1.7%) with multiple serotypes all covered by PCV15. The median age of the 31 patients with CAP due to serotypes 22F or 33F did not differ significantly from patients with PCV13-serotype CAP (62.0 years, IQR 53.6–78.0 years versus 71.7 years, IQR 60.0–81.0 years respectively, p = 0.11) (Table 3). Compared to people with PCV13-serotype CAP only, cerebrovascular disease was an independent risk factor for CAP with serotypes 22F/33F (aOR 7.97, 95% CI 2.16–29.5, p = 0.002) (Supplementary Table S4).

**Table 3:** Age and number of clinical risk factors for additional non-PCV13 serotypes in each vaccine class compared to single serotype/multiple PCV13-serotype CAP.

	PCV13 serotypes (reference)	PCV15-nonPCV13 serotypes	PCV20-nonPCV13 serotypes	PPV23-nonPCV13 serotypes	Non-vaccine serotypes
Total	257	31	192	252	86
Median age (IQR)	71.7 (60.0–81.0)	62.0 (53.6–.78.0)	**62.0 (50.6**–**72.0)**^b^	**64.5 (53.8**–**74.0)**^b^	71.0 (59.0–78.1)
≥1 clinical risk factor^a^ (%)	163 (63.4)	19 (61.3)	**102 (53.1)**^b^	146 (57.9)	59 (68.6)

Key: IQR inter-quartile range.

Each vaccine category includes single serotype detections, and multiple serotypes where the serotypes are all within the same vaccine categorisation.

a Clinical risk factors: chronic respiratory disease, chronic heart disease, chronic liver disease, chronic kidney disease, diabetes requiring treatment, immunosuppression (immunosuppressive medications, previous solid organ transplant, previous bone marrow transplant, splenic dysfunction, neoplastic disease with active treatment in last 6 months, haematological malignancy, primary immunodeficiency, HIV), CSF leak or cochlear implant.

b Bold values indicate a p-value of <0.05 compared to PCV13 serotypes.

### PCV20-nonPCV13 serotype pneumonia

PCV20-nonPCV13 serotypes were detected in 192 (24.6%, 95% CI 21.7–27.7%) patients with pneumococcal CAP. Patients with PCV20-nonPCV13 serotype CAP were younger than those with PCV13 serotype CAP (median age 62.0 years, IQR 50.6–72.0, versus 71.7 years, IQR 60.0–81.1 years, p < 0.001) (Table 3). The odds of PCV20-nonPCV13 serotype CAP were lower in males (aOR 0.47, 95% CI 0.39–0.57, p < 0.001) and in people who had previously reported receiving PPV23 vaccine (aOR 0.43, 95% CI 0.32–0.58, p < 0.001). Patients with PCV20-nonPCV13 serotype CAP had lower odds of readmission within 30 days compared to patients with PCV13 serotype CAP (aOR 0.38, 95% CI 0.17–0.89, p = 0.03). Compared to PCV13 serotype CAP, PCV20-nonPCV13 serotype CAP was significantly more likely in younger people aged 16–64 years and with no clinical risk factors (aOR 1.84 (95% CI 1.23–2.74), p = 0.003), and significantly less likely in those aged ≥65 years with one or more clinical risk factors (aOR 0.70, 95% CI 0.49–0.99, p = −0.05) (Supplementary Table S5). Results from the sensitivity analysis excluding serotype 8 are detailed in Appendix 2 of the online supplement.

### PPV23-nonPCV13-serotype pneumonia

PPV23 serotypes accounted for 74.8% (n = 584, 95% CI 71.6–77.7%) of pneumococcal CAP. People with PPV23-nonPCV13 serotype CAP (n = 252, 32.3%, 95% CI 29.1–35.6%) of the pneumococcal CAP cohort were significantly younger compared to those with PCV13-serotype CAP (median age 64.5 years, IQR 53.8–74.0 versus 71.7 years, IQR 60.0–81.0, p < 0.001) (Table 3). Decreased odds of PPV23-nonPCV13 serotype CAP was observed in males (aOR 0.52, 95% CI 0.33–0.81, p = 0.004), and in people who reported PPV23 vaccination (aOR 0.52, 95% CI 0.32–0.84, p = 0.007) (Supplementary Table S7).

## Discussion

We found that during the study period, pneumococcal pneumonia continued to account for a large proportion (40.7%) of hospitalised CAP with 31% and 35% of single serotype pneumococcal CAP due to serotypes covered by PCV13 and PCV15 respectively. This is despite multiple years of infant and adult pneumococcal vaccination programmes of relatively high coverage in the UK; 71.5% for adults ≥65 years and 90.5% for infants in 2019–2020.^16^^,^^17^

A PCV13 serotype (as a single serotype or with other serotypes) was implicated in almost half the patients diagnosed with pneumococcal pneumonia. This is in accordance with the findings of a systematic review of pneumococcal serotypes in people aged 50 years and above, in which PCV13 serotypes caused 49% of pneumococcal CAP cases in pooled studies from across Europe, North America and Asia in the period after the introduction of PCV10/13 into national childhood immunisation programmes.^10^ Males and people aged ≥65 years had greater odds of PCV13 serotype CAP compared to non-PCV13 serotype CAP. This substantial burden of PCV13-serotype pneumonia in adults remains despite PCV13 implementation for children since 2010 and previous assumptions that herd protection would have a large effect in indirectly protecting adults at high risk of pneumococcal disease.^18^^,^^19^ In 2015, the UK Joint Committee on Vaccination and Immunisation advised that PCV13 vaccination for older people would not be cost-effective based on the assumption that the IPD and CAP incidence due to PCV7 serotypes would continue to decline across all age groups.^20^ This downward trend appears to have reversed during 2016/2017, one year prior to the start of this cohort study, being particularly driven by non-PCV13 serotypes alongside ongoing transmission of serotypes 3 and 19A,^9^^,^^11^ which were also the two most prevalent PCV13 serotypes in our cohort, accounting for 70.4% and 16.9% of PCV13 serotypes respectively. The shift away from PCV13 serotypes following the introduction of infant PCV13 immunisation has been less marked than the shift seen after PCV7 was introduced, and may reflect lower vaccine efficacy against particular PCV13 serotypes and the persistence of serotype 3. Several studies have indicated that the indirect protective effects of childhood PCV13 immunisation against IPD in adults may be lower for serotype 3.^21^, ^22^, ^23^, ^24^ With regards to direct PCV13 immunisation of adults, a systematic review (authored by the manufacturers of PCV13) which included three studies, reported vaccine effectiveness of 52.5% (95% CI 6.2–75.9%) against serotype 3 hospitalised CAP in adults aged 65 years and over.^25^ However, the population-level impact of direct PCV13 immunisation on IPD and pneumonia caused by serotype 3 remains uncertain, with reported increased carriage rates and no decrease in invasive disease since the implementation of PCV13 in adults in the United States.^26^

Direct adult PCV13 immunisation has not been recommended in the UK and it is likely that few, if any, patients in our study had received PCV13 as an adult. However, the JCVI “recently agreed that either PCV20 or PPV23 could be used for the adult pneumococcal programme.” “The Committee also indicated that PCV20 was likely to prevent more disease than PPV23 and waning of immunity may occur at a slower rate.”^27^ In our study a further 5% of pneumococcal CAP was attributed to the additional PCV15 serotypes and almost a quarter due to a unique PCV20 (non-PCV13) serotype. This is in accordance with the findings of other recent European studies,^28^, ^29^, ^30^ and suggests that potentially considerable numbers of pneumococcal CAP could be averted with the higher-valency vaccines.

After serotype 3, serotype 8 was one of the major contributors to pneumococcal CAP and the most prevalent serotype in bacteraemic patients. Since 2013/2014, the incidence of serotypes 8 and 12F IPD, which are not included in PCV13 nor PCV15 but are included in PCV20, has been increasing in the UK.^9^^,^^11^ A study from the southwest of the UK found that during a time period comparable to that of our study, the most common serotypes in patients with invasive pneumococcal disease were 8, 3, 9N, 19A and 19F,^31^ which were also the most commonly detected serotypes in our cohort of mostly non-invasive pneumococcal pneumonia patients. This broadly corresponds to the serotype distribution in IPD across Europe during 2018 where serotypes 8, 3, 19A, 22F and 12F were most prevalent following an increase in serotypes 8 and 3 during 2014–18.^32^

In our cohort, people under 65 years with no pneumococcal risk factors (and therefore not currently targeted for pneumococcal vaccination) were more likely to develop pneumonia with a PCV20-non13 serotype than other serotypes. When patients with serotype 8 pneumonia were excluded in the sensitivity analysis, the association became statistically non-significant, suggesting that the association may be driven by this particular serotype, although this finding should be interpreted with caution. A pneumococcal carriage study in children and their household contacts in England and Wales during 2015/2016 did not identify nasopharyngeal carriage with serotype 8.^33^ In contrast, another study, including healthy adults who did not have contact with young children and who were not normally included in colonisation studies, did detect carriage of serotypes with invasive potential, including serotype 8,^34^ suggesting that nasopharyngeal carriage patterns may be different in adults compared to children.

Almost three-quarters of pneumococcal CAP cases had a PPV23-serotype detected and the median time since PPV23 vaccination in our cohort was ten years. The effectiveness of PPV23 in preventing non-invasive pneumonia has been the subject of much debate.^35^ A review of data published from 2010 to 2020 concluded that PPV23 does confer some protection against vaccine-type pneumococcal pneumonia, but that effectiveness may be lower in older adults aged 75 years and over, those with certain underlying conditions, and in people vaccinated more than 5 years before disease onset.^36^ A more recent test-negative design study indicated that PPV23 vaccination may offer moderate (adjusted vaccine effectiveness 24%) long-term protection against hospitalisation with PPV23 serotype pneumonia for adults aged 65 years and over, but not for those who are 75 years and older.^7^ Although our study did not aim to assess the effectiveness of PPV23, our findings are consistent with these reports.

A strength of our study is that it is a large prospective multicentre study which describes the distribution of pneumococcal serotypes in patients hospitalised with CAP -in different parts of the UK. Use of the urinary Bio-Plex24 assay permitted the identification of serotypes not otherwise classifiable from other non-invasive specimens.

A major limitation of our study is that due to lack of power only very large associations may have been detected. The statistically significant results we observed are therefore likely to represent true associations. A further limitation is that the Bio-Plex24 assay is only able to detect 24 serotypes, hence the burden of pneumococcal CAP within the cohort may have been underestimated. Additionally, there are recognised cross-reactions in the Bio-Plex24 assay with serotypes which are not targeted in the assay, where the serotype was designated based on the predominant serotype using national IPD data and serotypes may vary in their predilection to cause invasive disease. This may have caused under-reporting of less common non-vaccine serotypes and thus underestimation of the prevalence of NVT pneumonia. For example, serotypes 11A and 16F cross-react but serotype 11A was reported as it was the more prevalent serotype in IPD during the study period; the absolute difference between these two serotypes was not large, up to 40% of serotype 11A reported in this manner may actually have been serotype 16F.

We observed that some patients only had a positive blood culture or BinaxNOW assay, so there may have been some underascertainment of pneumococcal pneumonia as these tests were not performed on samples from every patient, nor did we use sputum results to confirm pneumococcal aetiology. Another limitation is that some patients with pneumococcal pneumonia may have been misclassified if pneumococcal antigens were not detected in the BinaxNow and Bio-Plex24 assays; lack of urinary pneumococcal antigen may be due to early presentation or prior antibiotic treatment. Furthermore, although concordance between direct blood culture serotypes and urinary serotypes was high, there were some bacteraemic patients with mismatch between these two investigations. The high sensitivity of the Bio-Plex24 assay may, therefore, in some cases be detecting carriage rather than disease serotypes. In our current study, almost 20% of patients with pneumococcal CAP had multiple serotypes identified in the Bio-Plex24 assay, similar to our previous studies.^11^ Whether this is due to false positive results, including colonisation of the upper respiratory tract, or represents true infection with multiple serotypes remains uncertain. To our knowledge only one study has investigated multiple serotypes in non-invasive pneumococcal disease; a prospective cross-sectional study from Japan, which used nanofluidic real-time PCR to identify pneumococcal serotypes in sputum, reported a high prevalence (42%) of multiple serotypes in adult patients with pneumonia, with lower risk of multiple serotype pneumonia in people vaccinated with PPV23 within the preceding five years.^37^ We did not observe this association in our adjusted analysis, but the significance of multiple serotypes in non-invasive pneumococcal disease warrants further investigation.

As people who did not consent to being studied were older and had more chronic health conditions than those who consented, it is likely that the sickest and frailest patients were underrepresented in our cohort. All the patients enrolled into this study were hospitalised with CAP, so the results may not be generalisable to non-hospitalised adults with less severe disease.

Our study was conducted prior to the SARS-CoV2 pandemic. The epidemiology of pneumococcal CAP was been influenced by social restrictions imposed during the pandemic. It will therefore be important to monitor changes in pneumococcal serotype distribution in the post-pandemic years. PCV15 and PCV20 vaccines have now been approved for use in children by regulatory authorities in the United, States, Europe and the UK (Food and Drug Administration, European Medicines Agency, Medicine and Healthcare products Regulatory Agency) and since 2022 have been recommended in many European countries,^38^ further reinforcing the importance of monitoring shifts in pneumococcal serotypes in future studies.

In conclusion, we report that the burden of PCV13 serotype pneumococcal pneumonia remains substantial in the adult population several years after the infant PCV13 immunisation programme, and that the risk is greatest in older people and those with high-risk comorbidities. In view of these findings, the use of newer, higher valency PCVs should be considered for older people; there are several novel pneumococcal vaccines under evaluation, including a 21-valent PCV which has a different set of serotypes to PCV13, 24-valent vaccines and a PCV with more than 30 serotypes. Monitoring for serotype replacement will be required following any introduction of new (higher valency) pneumococcal vaccines.

## Contributors

WSL, CS, NF and AH were responsible for study conception and design. HL, SA, AH, HP, VB, RCE-P, LB, DA, JB, PD, TB, CR, DL and SE were responsible for data acquisition. TM, LL and CT were responsible for the statistical analysis. LL and WSL drafted the initial version of the article. All authors contributed to data interpretation and read, commented on and approved the final version of the article.

## Data sharing statement

The data are available from the corresponding author upon reasonable request.

## Declaration of interests

WSL reports unrestricted investigator-initiated research funding from Pfizer to WSL's institution. WSL is Chair of COVID-19 Immunisation, Joint Committee of Vaccination and Immunisation (JCVI) (unpaid), is an unpaid expert panel member for NICE COVID-19 Guidelines, and is an unpaid member of the NHSE Specialist Commissioning Group for Covid-19 therapeutics. University of Nottingham paid costs to Liverpool University Foundation Hospital Trust for the completion of the field work; NF is an honorary member of LUHFT and of the department that received this funding. NF is PI on grants from Wellcome, MRC, EDTCP and Gates Foundation and is a co-investigator on awards from Seqirus CSl and from GSK for studies unrelated to this work, participates on 3 Data Safety Monitoring Boards for NIHR and MRC funded research trials in relation to pneumococcal challenge models outside the submitted work, and serves on the pneumococcal subcommittee of the JCVI UK. CT participated in a CMV vaccine advisory board meeting in May 2022, unrelated to the topic of this paper. SE declares participation in a Virtual Advisory Board for a pneumococcal project organised by Sanofi Pasteur SA unrelated to the submitted work. The UK Health Security Agency (UKHSA) received monies from the University of Nottingham from an unrestricted grant from Pfizer for this work. The Vaccine Preventable Bacteria Section of UKHSA has received grants from Pfizer for investigator led research unrelated to the current project. The UKHSA provides vaccine manufacturers (GlaxoSmithKline (GSK), MSD, Pfizer) with post marketing surveillance reports on vaccine-preventable disease, including pneumococcal infections for which a cost recovery charge is made and which is unrelated to the submitted work.

No other authors declare competing interests.
