# Supplementary material for: Pneumococcal serotypes and risk factors in adult community-acquired pneumonia 2018–20; a multicentre UK cohort study
Source: Lancet Reg Health Eur. 2023 Dec 11;37:100812. doi: 10.1016/j.lanepe.2023.100812 (PMC10758948; doi:10.1016/j.lanepe.2023.100812)
Supplement: Supplementary Tables and Figures [file mmc1.docx]

**Supplementary Tables and Figures**

**Pneumococcal serotypes and risk factors in adult community-acquired pneumonia 2018-20; a multicentre UK cohort study**

Louise Lansbury^1,2^ (PhD), Hannah Lawrence^1,2^ , Tricia M McKeever^1,2^ (PhD), Neil French^3,4^(PhD), Stephen Aston^3^, Adam T Hill^5^, Harry Pick^1,7^, Vadsala Baskaran^1,2^, Rochelle C Edwards-Pritchard^1,2^(MSci Hons), Lesley Bendall^2^, Deborah Ashton^2^, Jo Butler^7^, Priya Daniel^6^ (PhD), Thomas Bewick^6^, Chamira Rodrigo^7^, David Litt^8^(PhD), Seyi Eletu^8^(PhD), Carmen L. Sheppard^8^,(PhD), Norman K Fry^8,9^(PhD), Shamez Ladhani^9^, Caroline Trotter (PhD)^10^, Wei Shen Lim^2,7^(FRCP)

^1^Faculty of Medicine and Health Sciences, University of Nottingham, Nottingham, UK

^2^National Institute for Health Research (NIHR) Nottingham Biomedical Research Centre, UK

^3^Liverpool University Hospitals NHS Foundation Trust, Liverpool, UK

^4^Institute of Infection Veterinary & Ecological Science, University of Liverpool, UK

^5^Centre for Inflammation Research, University of Edinburgh

^6^Respiratory Medicine, University Hospitals of Derby and Burton NHS Foundation Trust, Derby, UK

^7^Department of Respiratory Medicine, Nottingham University Hospitals NHS Trust, Nottingham, UK

^8^Respiratory and Vaccine Preventable Bacteria Reference Unit, UK Health Security Agency, Colindale, UK

^9^Immunisation and Vaccine Preventable Diseases, UK Health Security Agency, Colindale, UK

^10^Disease Dynamics Unit, Department of Veterinary Medicine, University of Cambridge, Cambridge, UK

Contents

[Appendix 1 METHODS 3](#_Toc151039018)

[Appendix 2 RESULTS 4](#_Toc151039019)

[Supplementary Table 1 Association between clinical risk factors and multiple pneumococcal serotypes 5](#_Toc151039020)

[Supplementary Table 2 Association between clinical risk group and multiple serotypes 7](#_Toc151039021)

[Supplementary Table 3: Blood culture pneumococcal serotype designations which were discrepant with urinary Bioplex24 assay serotypes 8](#_Toc151039022)

[Supplementary Table 4 Clinical features and comparative analysis of additional PCV15 serotypes (22F and 33F) versus PCV13 serotype CAP 9](#_Toc151039023)

[Supplementary Table 5 Clinical features and comparative analysis of additional PCV20 -non PCV13 serotype*s* versus PCV13 serotype CAP 11](#_Toc151039024)

[Supplementary Table 6 Sensitivity analysis of PCV20-nonPCV13 serotypes CAP excluding serotype 8 13](#_Toc151039025)

[Supplementary Table 7 Association between clinical risk factors and PPV23-nonPCV13 serotypes versus PCV13 serotype CAP 16](#_Toc151039026)

[Supplementary Figure 1 18](#_Toc151039027)

[Supplementary Figure 2 19](#_Toc151039028)

[Supplementary Figure 3 20](#_Toc151039029)

# Appendix 1 METHODS

Acute admissions were screened for study eligibility each weekday and reviewed within 48 hours of admission by the study team. Eligibility for inclusion in the study was defined as patients aged ≥16 years presenting with ≥1 symptoms associated with a lower respiratory tract infection (cough, increasing dyspnoea, sputum production and/or fever), with acute abnormalities consistent with infection on a chest radiograph taken within 48 hours of admission, and treated as CAP. Exclusion criteria were hospitalisation within ten days of the index admission, or diagnosis of post-obstructive pneumonia secondary to lung cancer. For this analysis, only data for patients admitted prior to 10 March 2020 were included, as this date marks the start of the SARS-CoV-2 pandemic period. Informed consent was sought from all eligible participants. For patients lacking capacity, proxy consent was sought from their personal representative. A standard proforma was used to collect demographic and clinical information through direct questioning of the patient or their representative, medical notes review, and electronic data resources for imaging, biochemistry and microbiology results. Readmission and mortality data were collected from electronic resources up to 30 days following discharge.

**Bio-Plex 24 Assay testing**

Urine aliquots from each site were frozen at -70C and batch transported to the Respiratory and Vaccine Preventable Bacteria Reference Unit, UK Health Security Agency (formerly Public Health England) for pneumococcal serotyping using a validated multiplex immunoassay (Bio-Plex24). The Bio-Plex24 assay uses human monoclonal antibodies (mAbs) to detect the pneumococcal serotypes 1, 2, 3, 4, 5, 6A, 6B, 7F, 8, 9N, 9V, 10A, 11A, 12F, 14, 15B, 17F, 18C, 19A, 19F, 20, 22F, 23F, 33F and pneumococcal cell-wall polysaccharide.^[[1]](#footnote-2)^ As 16 of the mAbs used in the Bio-Plex24 assay exhibit a degree of cross-reactivity with non-targeted pneumococcal serotypes, a checkerboard system was used to interpret results and allow identification. Where alternative serotypes were reported, the final serotype designation was based upon the predominant serotype observed in national IPD surveillance data for the corresponding time-period using a probabilistic approach.

**Statistical Analysis**

The percentage of missing values ranged from 0.05% for age up to 16.9% for neoplasia, and only 72% of the 1921 patients in the cohort would have been available for analysis under the traditional listwise deletion method. We addressed the problem of missing data using the multiple imputation technique including all analysis variables for each outcome under the assumption that data are missing at random. Stata 17’s ‘mi impute chained’ command generated five imputed datasets. Analyses run on each dataset were pooled according to Rubin’s rules. Imputed values compared reasonable to observed values and results using listwise deletion are similar to MI so imputed results are presented.

The following variables were included in the multiple imputation models: outcome, age, smoker, gender, ppv23 vaccine receipt, residential care, neoplasia, liver disease, cerebrovascular disease, diabetes, chronic lung disease, chronic heart disease, immunosuppression, chronic renal disease, cognitive impairment, ischaemic heart disease, 30-day mortality, 30-day readmission, critical care

# Appendix 2 RESULTS

**Multiple serotypes**

Of the 721 patients with a positive Bio-Plex24 result, 52 were non-typeable (6·8% of all assays), Four patients had an equivocal result and were excluded from the serotype analyses. A single serotype was identified in the Bio-Plex24 assay in 522 of the 760 (68·7%) tested patients, and multiple serotypes were detected in 149 (19·6%); two serotypes were identified in 118 (15·5%) tested patients, and three or more serotypes in 31 (4·1%). Forty-two (28·2%) people with multiple serotypes had both serotypes 3 and 8, and thirteen (8·7%) had serotype 5 with serogroup 15 (Supplementary Figure 2). Patients with multiple serotypes were more likely to be older (median age 72·5 years, IQR 60·0–82·0 years versus 69·0 years, IQR 56·0–78·4 years, p=0·005) and were more likely to be readmitted within 30 days (aOR 1·48, 95% CI 1·16 – 1·88, p=0·001) (Supplementary Table 1) On adjusted analysis an association was observed between multiple serotypes and age greater than 65 years with no other risk factors (aOR 1,27, 95% CI 1·05 – 1·55, p=0·02, and also being in the pneumococcal clinical risk group (aOR 1·13, 95% CI 1·05 – 1·23, p=0·002. (Supplementary Table 2)

**Bio-Plex24 and Blood Culture pneumococcal serotyping results**

Blood cultures were performed in 1394 patients (72·6%), with *S.pneumoniae* detected in 78 patients (5·6%). A blood culture serotype was available for 70 of 78 bacteraemic patients (90%). Seventeen different serotypes were identified, with serotype 8 being the most prevalent (35·8%), followed by serotypes 3 (14·9%) and 9N (11·9%) (Supplementary Figure 3). Of the 59 patients with both a blood culture serotype and Bio-Plex24 result, the same serotypes were identified in 47 patients (79·7%), with 43 (93·6%) of these having a single serotype detected by Bio-Plex24. A different serotype was detected in blood and Bio-Plex24 in five patients (8·5%) and a further five patients had blood culture serotypes (16F, 24F, 35B and 13) not targeted in the Bio-Plex24 assay, hence a “non-typeable” result was generated (Supplementary Table 3). ·Two patients (3·4%) with a blood culture serotype (3 and 23A) had a negative Bio-Plex24 result.

**Sensitivity analysis excluding serotype 8**

When 123 patients with serotype 8 were excluded, the point estimates for several risk factors changed by >10% and the observed associations with PPV23 vaccine receipt was no longer statistically significant (aOR 0·68, 95% CI 0·44–1·04, p=0·08 )(Supplementary Table 6). For people aged 16–64 years with no risk factors, the adjusted point estimate decreased by 16% and was no longer statistically significant (aOR 1·53, 95% CI 0·76–3·06, p=0·23). For people ≥65 years with one or more clinical risk factor, the point estimate was unchanged and remained statistically significant when patients with serotype 8 were excluded from the analyses (aOR 0·70, 95% CI 0·37–2·07, p=<0·001) (Supplementary Table 6).

# Supplementary Table 1 Association between clinical risk factors and multiple pneumococcal serotypes

|  | **Multiple STs (149)** | **Single serotype**  **(571)** | **OR (95% CI)** | **p-value** | **aOR*** | **p-value** |
| --- | --- | --- | --- | --- | --- | --- |
| **Age (IQR)** | 72·5 (60·0-82·0) | 69·0 (56·0-78·4) |  | **0·005** |  |  |
| **16-49y**  **50-65y**  **65-574y**  **75-84y**  **≥85y** | 15 (10·0)  33 (22·1)  34 (22·8)  41 (27·5)  25 (16·8) | 92 (16·1)  149 (26·1)  143 (25·0)  119 (20·8)  68 (11·9) | Ref  1·36 (0·70-2·64)  1·46 (0·75-2·82)  2·11 (1·10-4·05)  2·25 (1·10-4·60) | 0·005 |  |  |
| **Male** | 83 (55·7) | 298 (52·2) | 1·15 (0·80-1·65) | 0·46 | 1·18 (0·89-1·57) | 0·24 |
| **Care home** | 0 | 10 (1·7) | - |  |  |  |
| **Ppv23 receipt** | 68 (54·4) | 220 (42·5) | 1·47 (1·00-2·13) | **0·04** | 1·54 (0·92-2·59) | 0·10 |
| **Smoking** |  |  |  |  |  |  |
| **Never**  **Ex**  **Current** | 44 (30·3)  70 (48·3)  31 (21·4) | 143 (25·4)  279 (49·6)  141 (25·0) | Ref  0·82 (0·53-1·25)  0·71 (0·43-1·20) | 0·19† |  |  |
| **Malignancy** | 10 (6·7) | 40 (7·0) | 0·85 (0·43-4·66) | 0·64 | 0·92(0·48-1·78) | 0·81 |
| **Liver disease** | 5 (3·3) | 12 (2·1) | 1·61 (0·56-4·72) | 0·37 | 2·16 (1·50-3·11) | **<0·001**· |
| **Chronic kidney disease** | 15 (10·0) | 66 (11·5) | 0·93 (0·52-1·67) | 0·81 | 0·82 (0·63-1·06) | 0·13 |
| **Chronic heart disease** | 29 (19·4) | 114 (19·9) | 0·97 (0·61-1·53) | 0·89 | 0·73 (0·63-0·86) | **<0·001** |
| **CCF** | 10 (6·7) | 45 (7·8) | 0·85 (0·42-1·73) | 0·66 | 0·72 (0·24-2·17) | 0·56 |
| **Ischaemic heart disease** | 25 (16·8) | 87 (15·2) | 1·14 (0·70-1·86) | 0·60 | 2·14 (0·99-4·58) | 0·05 |
| **Chronic lung disease** | 61 (40·9) | 212 (37·1) | 1·18 (0·81-1·71) | 0·37 | 1·07 (0·73-1·58) | 0·72 |
| **COPD** | 54 (36·2) | 186 (32·5) | 1·18 (0·81-1·72) | 0·40 | 1·09 (0·72-1·65) | 0·67 |
| **Asthma** | 16 (10·7) | 84 (14·7) | 0·70 (0·40-1·23) | 0·21 | 0·71 (0·40-1·28) | 0·25 |
| **Diabetes** | 27 (18·1) | 101 (17·7) | 1·03 (0·64-1·65) | 0·90 | 1·04· (0·87-1·23) | 0·67 |
| **Cerebrovascular disease** | 13 (8·7) | 36 (6·3) | 1·43 (0·735-2·80) | 0·29 | 1·32 (0·85-2·04) | 0·22 |
| **Cognitive impairment** | 5 (3·3) | 17 (2·9) | 1·14 (0·42-3·09) | 0·80 | 0·91 (0·53-1·55) | 0·35 |
| **Immunosuppression** | 5 (3·3) | 31 (5·4) | 0·60 (0·23-1·58) | 0·31 | 0·58 (0·47-0·71) | **<0·001** |
| **Number of clinical risk factors**  **0**  **1**  **2**  **≥3** | 51 (34·2)  66 (44·3)  25 (16·7)  7 (4·7) | 221 (38·7)  209 (36·6)  105 (18·4)  36 (6·3) | Ref  1·37 (0·91-2·06)  1·03 (0·61-1·76)  0·84 (0·35-2·00) | 0·97† | Ref  1·27 (0·79-2·02)  0·89 (0·49-1·64)  0·83 (0·32-2·17) | 0·71† |
| **CURB score**  **0-1 (low)**  **2 (moderate)**  **≥3 (high)** | 72 (48·3)  45 (30·2)  32 (21·5) | 293 (51·3)  164 (28·7)  114 (19·9) | Ref  1·11 (0·73-1·70)  1·14 (0·71-1·83) | 0·53† | Ref  0·94 (0·54-1·64)  0·91 (0·54-1·64) | 0·38† |
| **Critical care admission** | 6 (4·0) | 47 (8·2) | 0·47 (0·20-1·12) | 0·09 | 0·52 (0·34-0·79) | **0·002** |
| **30-day mortality** | 11 (7·4) | 39 (6·8) | 1·09 (0·55-2·19) | 0·80 | 0·86 (0·71-1·05) | 0·15 |
| **30-day readmission** | 29 (19·5) | 81 (14·2) | 1·45 (0·91-2·34) | 0·12 | 1·48 (1·16-1·88) | **0·001** |

Key: STs serotypes; OR Odds ratio; aOR adjusted odds ratio; IQR Interquartile range CCF Congestive cardiac failure; COPD Chronic obstructive pulmonary disease

Data are presented as n (%). Bold values indicate p-value <0.05.

*Adjusted for age, gender, co-morbidities, PPV23 vaccine clustered by site, number in model = 720

†p-value for trend

# Supplementary Table 2 Association between clinical risk group and multiple serotypes

|  | **Multiple STS (149)** | **Single serotype or non-typeable**  **(571)** | **OR (95% CI)** | **p-value** | **aOR* (95% CI)** | **p-value** |
| --- | --- | --- | --- | --- | --- | --- |
| 16-64 years, no clinical risk factor | 23 (15·4) | 143 (25·0) | 0·55 (0·34-0·88) | **0·01** | 0·61 (0··51-0·73) | ·**<0·001** |
| 16-64 years, ≥1 risk factor | 25 (16·7) | 98 (17·2) | 0·97 (0·60-1·58) | 0·91 | 1·04 (0·84-1·31) | 0·68 |
| ≥65 years, no clinical risk factor | 25 (16·7) | 75 (13·1) | 1·33 (0·81-2·18) | 0·25 | 1·27 (1·05-1·55) | **0·02** |
| ≥65 years, ≥1 risk factor | 75 (50·3) | 255 (44·6) | 1·26 (0·87-1·80) | 0·22 | 1·09 (0·91-1·31) | 0·32 |
| Pneumococcal clinical risk group | 125 (86·5) | 428 (74·9) | 1·74 (1·08-2·80) | **0·02** | 1·13 (1·05-1·23) | **0·002** |

Key: ST serotypes

OR Odds ratio

aOR adjusted odds ratio

Data are presented as n (%). Bold values indicate p-value <0.05. Clinical risk factors include chronic respiratory disease, chronic heart disease, chronic liver disease, chronic kidney disease, diabetes requiring treatment, immunosuppression (immunosuppressive medications, previous solid organ transplant, previous bone marrow transplant, splenic dysfunction, neoplastic disease with active treatment in last 6 months, haematological malignancy, primary immunodeficiency, HIV), CSF leak or cochlear implant)

*Adjusted for gender, ppv23 vaccine, number in model = 720

†p-value for trend

# Supplementary Table 3: Blood culture pneumococcal serotype designations which were discrepant with urinary Bioplex24 assay serotypes

| **Blood culture pneumococcal serotype** | **Urinary Bioplex-24 pneumococcal serotype** | **Number of participants** |
| --- | --- | --- |
| 3 | Non-typeable* | 1 |
| 7C | Multiple (11A/C/E or 16A/F and 20) | 1 |
| 11A | 8 | 1 |
| 12F | 8 | 1 |
| 13 | Non-typeable | 1 |
| 16F | Non-typeable | 2 |
| 22F | Multiple (Group 15 and 17F) | 1 |
| 24F | Non-typeable | 1 |
| 35B | Non-typeable | 1 |
|  |  |  |
| 3 | Not detected | 1 |
| 23A | Not detected | 1 |

* A non-typeable result in the Bioplex-24 assay indicates that pneumococcal cell-wall polysaccharide was detected but an individual serotype result could not be generated.

# Supplementary Table 4 Clinical features and comparative analysis of additional PCV15 serotypes (22F and 33F) versus PCV13 serotype CAP

|  | **Additional PCV15 STs (n=31)** | **PCV13 (n=257)** | **OR (95% CI)** | **p-value** | **aOR*** | **p-value** |
| --- | --- | --- | --- | --- | --- | --- |
| **Median Age (IQR)** | 62·0 (53·6-78·0) | 71·7 (60·0-81·0) |  | **0·11** |  |  |
| **Male** | 16 (51·6) | 157 (61·0) | 0·65 (0·31-1·38) | 0·26 | 0·58 (0·39-0·87) | **0·01** |
| **Care home** | 0 | 6 (2·3) | - | - | - | - |
| **Ppv23 receipt** | 11 (35·5) | 113/228 (49·6) | 0·61 (0·28-1·33) | 0·21 | 0·41 (0·21-0·78) | **0·01** |
| **Smoking** |  |  |  |  |  |  |
| **Never**  **Ex**  **Current** | 5 (16·1)  19 (61·3)  7 (22·6) | 65/253 (25·7)  137/253 (54·2)  51/253 (20·1) | Ref  1·80 (0·64-5·94)  1·78 (0·53-5·95) | 0·35† |  |  |
| **Malignancy** | 1 (3·2) | 17 (6·6) | 0·49 (0·05-4·92) | 0·54 | 0·51 (0·04-7·24) | 0·61 |
| **Liver disease** | 1 (3·2) | 4 (1·5) | 2·01 (0·22-18·66) | 0·54 | 1·17 (0·13-10·28) | 0·89 |
| **Chronic kidney disease** | 3 (9·7) | 37 (14·4) | 0·62 (0·18-2·16) | 0·46 | 0·88 (0·15-5·27) | 0·89 |
| **Chronic heart disease** | 8 (25,8) | 54 (21·0) | 1·31 (0·55-3·08) | 0·54 | 1·41 (0·57-3·49) | 0·46 |
| **Chronic cardiac failure** | 3 (25·8) | 23 (8·9) | 1·08 (0·30-3·83) | 0·90 | (1·79 (0·67-4·75) | 0·24 |
| **Ischaemic heart disease** | 6 (19·4) | 38 (14·8) | 1·38 (0·53-3·60) | 0·51 | 0·95 (0·35-2·57) | 0·93 |
| **Chronic lung disease** | 13 (41·9) | 89 (34·6) | 1·36 (0·63-2·90) | 0·43 | 1·84 (0·41-8·32) | 0·43 |
| **COPD** | 13 (41·9) | 78 (30·4) | 1·66 (0·77-3·55) | 0·19 | 2·85 (0·52-15·70) | 0·23 |
| **Asthma** | 6 (19·4) | 36 (14·0) | 1·47 (0·56-3·84) | 0·43 | 1·25 (0·38-4·12) | 0·71 |
| **Diabetes** | 4 (12·9) | 52 (20·2) | 0·58 (0·20-1·74) | 0·34 | 0·55 (0·28-1·08) | 0·08 |
| **Cerebrovascular disease** | 7 (22·6) | 18 (7·0) | 3·87 (1·47-10·20) | **0·006** | 6·35 (3·35-12·03) | **<0·001** |
| **Cognitive impairment** | 0 | 9 (3·5) | - | - | - | - |
| **Immunosuppression** | 0 | 17 (6·6) | - | - | - | - |
| **CURB score**  **0-1 (low)**  **2 (moderate)**  **≥3 (high)** | 19 (61·3)  6 (19·4)  6 (19·4) | 115 (44·7)  78 (30·4)  64 (24·9) | Ref  0·46 (0·18-1·22)  0·57 (0·22-1·49) | 0·15† | Ref  0·56 (0·16-1·99)  0·64 (0·16-2·60) | 1·00† |
| **Critical care admission** | 2 (6·4) | 21 (8·2) | 0·78 (0·17-3·48) | 0·74 | 0·76 (0·38-1·52) | 0·44 |
| **30-day mortality** | 1 (3·2) | 25 (9·7) | 0·30 (0·04-2·32) | 0·25 | 0·27 (0·09-0·78) | **0·02** |
| **30-day readmission** | 2 (6·4) | 44 (17·1) | 0·30 (0·07-1·32) | 0·11 | 0·25 (0·07-0·91) | **0·04** |
|  |  |  |  |  |  |  |
| **Subgroup analyses for specific risk groups** | | | | | | |
| 16-64 years, no clinical risk factor | 10 (32·2) | 47 (18·3) | 2·13 (0·94-4·82) | 0·07 | 1·72 (0·62-4·75) | 0·29 |
| 14-64 years, , ≥1 risk factor | 7 (22·6) | 40 (15·6) | 1·58 (0·64-3·92) | 0·32 | 1·16 (0·19-7·07) | 0·87 |
| ≥65 years, no clinical risk factor | 2 (6·4) | 45 (17·5) | 0·32 (0·07-1·41) | 0·13 | 0·35 (0·11-1·08) ^a^ | 0·07 |
| ≥65 years, ≥1 risk factor | 12 (38·7)) | 124(48·2) | 0·68 (0·32-1·45) | 0·32 | 0·95 (0·72-1·24) | 0·69 |
| Pneumococcal clinical risk group^‡^ | 21 (67·7) | 209 (81·3) | 0·90 (0·42-1·93) | 0·78 | 1·04 (0·40-2·76) | 0·93 |

STs serotypes; OR Odds ratio; aOR adjusted odds ratio; IQR Interquartile range CCF Congestive cardiac failure; COPD Chronic obstructive pulmonary disease

Data are presented as n (%). Bold values indicate p-value <0.05.

*Adjusted for age, gender, residential care, co-morbidities, ppv23 vaccine clustered by site, number in model = 288

†p-value for trend

Data on PPV23-receipt and smoking status available for 643 and 708 participants respectively

‡Pneumococcal clinical risk group includes everyone aged ≥65 years, plus anyone aged 16-64 years with one or more of the following clinical risk factors: chronic respiratory disease, chronic heart disease, chronic liver disease, chronic kidney disease, diabetes requiring treatment, immunosuppression (immunosuppressive medications, previous solid organ transplant, previous bone marrow transplant, splenic dysfunction, neoplastic disease with active treatment in last 6 months, haematological malignancy, primary immunodeficiency, HIV), CSF leak or cochlear implant)

^a^Adjusted for gender, residential care, ppv23 vaccine receipt

# Supplementary Table 5 Clinical features and comparative analysis of additional PCV20 -non PCV13 serotype*s* versus PCV13 serotype CAP

|  | | **Serotypes in PCV20-nonPCV13 (n=192)** | **PCV13 serotypes (n=257)** | **OR (95% CI)** | **p-value** | **aOR* (95% CI)** | **p-value** |
| --- | --- | --- | --- | --- | --- | --- | --- |
| **Baseline characteristics** | | | | | | | |
| **Median Age (IQR)** | | 62·0 (50·6-72·0) | 71·7 (60·0-81·0) |  | **<0·001** |  |  |
| **Gender, male** | | 85 (44·3) | 157 (61·0) | 0·47 (0·34-0·72) | **<0·001** | 0·47 (0·39-0·57) | **<0·001** |
| **Residential care** | | 1 (0·52) | 6 (2·3) | 0·19 (0·02-1·48) | 0·11 | 0·30 (0·07-1·20) | 0·09 |
| **PPV23 vaccine receipt** | | 55/174 (31·6) | 113/228 (49·6) | 0·43 (0·28-0·66) | **<0·001** | 0·43 (0·32-0·58) | **<0·001** |
| **Smoking**  **Never**  **Ex**  **Current** | | 49/190 (25·8)  81/190 (42·6)  60/190 (31·6) | 65/253 (25·7)  137/253 (54·2)  51/253 (20·1) | Ref  0·78 (0·49-1·24)  1·56 (0·92-2·64) | 0·09† |  |  |
| **Malignancy** | | 13 (6·8) | 17 (6·6) | 0·93 (0·45-1·92) | 0·84 | 0·93 (0·59 -1·46) | 0·76 |
| **Liver disease** | | 4 (2·1) | 4 (1·5) | 1·34 (0·33-5·45) | 0·68 | 1·36 (0·65-2·85) | 0·42 |
| **Chronic kidney disease** | | 15 (7·8) | 37 (14·4) | 0·50 (0·27-0·94) | **0·03** | 0·61 (0·39-0·98) | **0·04** |
| **Chronic heart disease** | | 32 (16·7) | 54 (21·0) | 0·75 (0·46-1·22) | 0·25 | 1·14 (0·95-1·35) | 0·15 |
| **Chronic cardiac failure** | | 13 (6·8) | 23 (8·9) | 0·73 (0·36-1·49) | 0·39 | 1·25 (0·37-4·28) | 0·72 |
| **Ischaemic heart disease** | | 27 (14·1) | 38 (14·8) | 0·94 (0·55-1·60) | 0·82 | 2·21 (1·32-3·67) | **0·002** |
| **Chronic lung disease** | | 64 (33·3) | 89 (34·6) | 0·94 (0·63-1·40) | 0·76 | 1·35 (0·92-1·96) | 0·12 |
| **COPD** | | 58 (30·2) | 78 (30·4) | 0·99 (0·66-1·49) | 0·97 | 1·43 (1·04-1·96) | **0·03** |
| **Asthma** | | 31 (16·1) | 36 (14·0) | 1·18 (0·70-1·99) | 0·53 | 1·01 (0· 87-1·18) | 0·87 |
| **Diabetes** | | 33 (17·2) | 52 (20·2) | 0·81 (0·50-1·32) | 0·40 | 0·85 (0·37-1·93) | 0·69 |
| **Cerebrovascular disease** | | 14 (7·3) | 18 (7·0) | 1·04 (0·50-2·16) | 0·91 | 1·71 (1·19-2·46) | **0·004** |
| **Cognitive impairment** | | 5 (2·6) | 9 (3·5) | 0·73 (0·24-2·23) | 0·58 | 1·18 (0·51-2·75) | 0·69 |
| **Immunosuppression** | | 7 (3·6) | 17 (6·6) | 0·53 (0·22-1·31) | 0·17 | 0·82 (0·59-1·14) | 0·23 |
| **Number of clinical risk factors**  **0**  **1**  **2**  **≥3** | | 90 (46·9)  63 (32·8)  27 (14·1)  12 (6·2) | 94 (36·5)  96 (37·4)  49 (19·1)  18 (7·0) | Ref  0·68 (0·45-1·05)  0·58 (0·33-1·00)  0·70 (0·32-1·53) | **0·05**† | Ref  0·75 (0·46-1·22)  0·78 (0·42-1·48)  1·07 (0·44-2·61) | 0·55† |
| **Disease Severity** | | | | | | | |
| **CURB score**  **0-1 (low)**  **2 (moderate)**  **≥3 (high)** | | 110 (57·3)  59 (30·7)  23 (12·0) | 115 (44·7)  78 (30·4)  64 (24·9) | Ref  0·79 (0·52-1·21)  0·38 (0·22-0·65) | **<0·001**† | Ref  1·09 (0·61-1·93)  0·55 (0·26-1·15) | 0·45† |
| **Critical care admission** | | 15 (7·8) | 21 (8·2) | 0·95 (0·48-1·90) | 0·89 | 0·91 (0·58-1·42) | 0·67 |
| **30-day mortality** | | 11 (5·7) | 25 (9·7) | 0·56 (0·27-1·17) | 0·12 | 0·74 (0·50-1·10) | 0·14 |
| **30-day readmission** | | 15 (7·8) | 44 (17·1) | 0·40 (0·21-0·74) | **0·004** | 0·38 (0·17-0,89) | **0·03** |
|  | |  |  |  |  |  |  |
| **Subgroup analyses for specific risk groups** | | | | | | | |
| 16-64 years, no clinical risk factor | | 71 (37·0) | 47 (18·3) | 2·62 (1·70-4·03) | **<0·001** | 1·84 (1·23-2·74)^a^ | **0·003** |
| 16-64 years, ≥1 risk factor | 41 (21·4) | | 40 (15·6) | 1·47 (0·91-2·39) | 0·12 | 1·25 (0·61-2·56)^a^ | 0·54 |
| ≥65 years, no clinical risk factor | 19 (9·2) | | 45 (17·5) | 0·52 (0·29-0·92) | **0·02** | 0··60 (0·29-1·23)^a^ | 0·16 |
| ≥65 years, ≥1 risk factor | 61 (31·8) | | 124(48·2) | 0·50 (0·34-0·74) | **<0·001** | 0·70 (0·49-1·00)^a^ | **0·05** |
| Pneumococcal clinical risk group^‡^ | 121 (63·0) | | 209 (81·3) | 0·64 (0·44-0·94) | **0·02** | 0·83 (0·57-1·21)^a^ | 0·33 |

Key: ST serotypes

OR Odds ratio

aOR adjusted odds ratio

Data are presented as n (%). Bold values indicate p-value <0·05.

*Adjusted for age, gender, residential care, ppv23 vaccine receipt, comorbidities clustered by site, number in model = 449

^a^Adjusted for gender, residential care, ppv23 vaccine receipt

‡Pneumococcal clinical risk group includes everyone aged ≥65 years, plus anyone aged 16-64 years with one or more of the following clinical risk factors: chronic respiratory disease, chronic heart disease, chronic liver disease, chronic kidney disease, diabetes requiring treatment, immunosuppression (immunosuppressive medications, previous solid organ transplant, previous bone marrow transplant, splenic dysfunction, neoplastic disease with active treatment in last 6 months, haematological malignancy, primary immunodeficiency, HIV), CSF leak or cochlear implant

†p-value for trend

# Supplementary Table 6 Sensitivity analysis of PCV20-nonPCV13 serotypes CAP excluding serotype 8

|  | **PCV20non13 (n=192) versus PCV13 CAP (n=257)** | | **PCV20nonPCV13 excluding serotype 8 (n=69) versus PCV13 CAP (n=257)** | | **% change in aOR point estimate** |
| --- | --- | --- | --- | --- | --- |
|  | **aOR* (95% CI)** | **p-value** | **aOR*** | **p-value** |  |
| **Baseline characteristics** | | | | | |
| **Gender, male** | 0·47 (0·39-0·57) | **<0·001** | 0·53 (0·41-0·68) | **<0·001** | **+13** |
| **PPV23 vaccine receipt** | 0·43 (0·32-0·58) | **<0·001** | 0·68 (0·44-1·04) | 0·08 | **+58** |
| **Malignancy** | 0·93 (0·59 -1·46) | 0·76 | 1·18 (0·40-3·44) | 0·76 | **+27** |
| **Liver disease** | 1·36 (0·65-2·85) | 0·42 | 3·14 (1·32-7·50) | **0·01** | **+130** |
| **Chronic kidney disease** | 0·61 (0·39-0·98) | **0·04** | 0·74 (0·31-1·76) | 0·50 | **+21** |
| **Chronic heart disease** | 1·14 (0·95-1·35) | 0·15 | 0·78 (0·52-1·17) | 0·23 | **-31** |
| **Chronic cardiac failure** | 1·25 (0·37-4·28) | 0·72 | 2·09 (1·14-3·84) | **0·02** | **+67** |
| **Ischaemic heart disease** | 2·21 (1·32-3·67) | **0·002** | 1·48 (0·42-5·06) | 0·54 | **-33** |
| **Chronic lung disease** | 1·35 (0·92-1·96) | 0·12 | 0·92 (0·40-2·15) | 0·85 | **-31** |
| **COPD** | 1·43 (1·04-1·96) | **0·03** | 1·10 (0·41-2·96) | 0·85 | **-23** |
| **Asthma** | 1·01 (0· 87-1·18) | 0·87 | 0·78 (0·64-0·96) | 0·02 | --23 |
| **Diabetes** | 0·85 (0·37-1·93) | 0·69 | 0·75 (0·32-1·72) | 0·49 | **-12** |
| **Cerebrovascular disease** | 1·71 (1·19-2·46) | **0·004** | 2·46 (1·49-4·30) | **0·002** | **+43** |
| **Cognitive impairment** | 1·18 (0·51-2·75) | 0·69 | 0·48 (0·31-0·77) | **0·002** | **+59** |
| **Immunosuppression** | 0·82 (0·59-1·14) | 0·23 | 0·80 (0·45-1·42) | 0·43 | **-2** |
| **CURB score** |  |  |  |  |  |
| **0-1 (low)**  **2 (moderate)**  **≥3 (high)** | Ref  1·09 (0·61-1·93)  0·55 (0·26-1·15) | 0·45†**†** | Ref  1·09 (0·50-2·41)  0·53 (0·19-1·51) | 0·35†**†** | 0  -4 |
| **Critical care admission** | 0·91 (0·58-1·42) | 0·67 | 0·85 (0·62-1·18) | 0·33 | -6 |
| **30-day mortality** | 0·74 (0·50-1·10) | 0·14 | 0·49 (0·26-0·90) | **0·02** | -34 |
| **30-day readmission** | 0·38 (0·17-0,89) | **0·03** | 0·59 (0·26-1·32) | 0·20 | **+55** |
|  |  |  |  |  |  |
| **Subgroup analysis of specific risk groups** | | | | | |
| 16-64 years, no clinical risk factor | 1·84 (1·23-2·74)^a^ | **0·003** | 1·53 (0·76-3·06)^a^ | 0·23 | **-16** |
| 16-64 years, ≥1 risk factor | 1·25 (0·61-2·56)^a^ | 0·54 | 1·35 (0·42-4·34)^a^ | 0·61 | +8 |
| ≥65 years, no clinical risk factor | 0··60 (0·29-1·23)^a^ | 0·16 | 0·75 (0·30-1·86)^a^ | 0·53 | **+25** |
| ≥65 years, ≥1 risk factor | 0·70 (0·49-1·00)^a^ | **0·05** | 0·70 (0·60-0·81)^a^ | **<0·001** | 0 |
| Pneumococcal clinical risk group^‡^ | 0·83 (0·57-1·21)^a^ | 0·33 | 0·87 (0·37-2·07)^a^ | 0·75 | **+5** |

Key: ST serotypes

OR Odds ratio

aOR adjusted odds ratio

Bold values indicate p-value <0.05 and change in point estimate of >10%

‡Pneumococcal clinical risk group includes everyone aged ≥65 years, plus anyone aged 16-64 years with one or more of the following clinical risk factors: chronic respiratory disease, chronic heart disease, chronic liver disease, chronic kidney disease, diabetes requiring treatment, immunosuppression (immunosuppressive medications, previous solid organ transplant, previous bone marrow transplant, splenic dysfunction, neoplastic disease with active treatment in last 6 months, haematological malignancy, primary immunodeficiency, HIV), CSF leak or cochlear implant)

*Adjusted for age, gender, residential care, co-morbidities, ppv23 vaccine clustered by site

^a^Adjusted for gender, residential care, ppv23 vaccine receipt

†**†**p-value for trend

# Supplementary Table 7 Association between clinical risk factors and PPV23-nonPCV13 serotypes versus PCV13 serotype CAP

|  | **PPV23- nonPCV13 (n=252)** | **PCV13 STs (n=257)** | **OR (95% CI)** | **p-value** | **aOR*** | **p-value** |
| --- | --- | --- | --- | --- | --- | --- |
| **Baseline characteristics** |  |  |  |  |  |  |
| **Age (IQR)** | 64·5 (53·8-74·0) | 71·7 (60·0-81·0) |  | **<0·001** |  |  |
| **Male** | 111 (44·0) | 157 (61·0) | 0·49 (0·34-0·70) | **<0·001** | 0·46 (0·40-0·51) | **<0·001** |
| **Care home** | 2 (0·8) | 6 (2·3) | 0·29 (0·06-1·42) | 0·13 | 0·44 (0·08-2·39) | 0·34 |
| **PPV23 receipt** | 78/227 (34·4) | 113/228 (49·6) | 0·50 (0·34-0·74) | **<0·001** | 0·49 (0·38-0·64) | **<0·001** |
| **Smoking** |  |  |  |  |  |  |
| **Never**  **Ex**  **Current** | 65/247 (26·3)  113/247 (45·7)  71/247 (31·3) | 65/253 (25·7)  137/253 (54·2)  51/253 (20·1) | Ref  0·82 (0·54-1·26)  1·39 (0·85-2·29) | 0·21† |  |  |
| **Malignancy** | 18 (7·1) | 17 (6·6) | 0·82 (0·37-1·83) | 0·62 | 0·74 (0·28-1·97) | 0·55 |
| **Liver disease** | 7 (2·8) | 4 (1·5) | 1·81 (0·52-6·25) | 0·35 | 1·89 (0·76-4·66) | 0·17 |
| **Chronic kidney disease** | 21 (8·3) | 37 (14·4) | 0·53 (0·30-0·94) | **0·03** | 0·62 (0·32-1·21) | 0·16 |
|  |  |  |  |  |  |  |
| **Chronic heart disease** | 43 (17·1) | 54 (21·0) | 0·77 (0·50-1·21) | 0·26 | 1·07 (0·99-1·16) | 0·08 |
| **Chronic cardiac failure** | 16 (6·3) | 23 (8·9) | 0·68 (0·35-1·33) | 0·26 | 1·11 (0·32-3·83) | 0·87 |
| **Ischaemic heart disease** | 35 (13·9) | 38 (14·8) | 0·93 (0·57-1·53) | 0·77 | 1·54 (0·71-3·34) | 0·28 |
| **Chronic lung disease** | 91 (36·1) | 89 (34·6) | 1·06 (0·74-1·53) | 0·74 | 1·46 (0·92-2·23) | 0·11 |
| **COPD** | 80 (31·7) | 78 (30·4) | 1·07 (0·73-1·55·) | 0·73 | 1·08 (0·75-1·58) | 0·66 |
| **Asthma** | 39 (15·5) | 36 (14·0) | 1·12 (0·69-1·84) | 0·64 | 0·99(0·86-1·14) | 0·90 |
| **Diabetes** | 45 (17·8) | 52 (20·2) | 0·86 (0·55-1·34) | 0·50 | 0·81 (0·39-1·69) | 0·58 |
| **Cerebrovascular disease** | 16 (6·3) | 18 (7·0) | 0·90 (0·45-1·81) | 0·77 | 1·29 (0·95-1·74) | 0·10 |
| **Cognitive impairment** | 7 (2·8) | 9 (3·5) | 0·75 (0·28-2·06) | 0·58 | 1·12 (0·36-3·49) | 0·84 |
| **Immunosuppression** | 11 (4·4) | 17 (6·6) | 0·64 (0·30-1·40) | 0·27 | 1·02 (0·64-1·59) | 0·97 |
| **Disease Severity** | | | | | | |
| **CURB score** |  |  |  |  |  |  |
| **0-1 (low)**  **2 (moderate)**  **≥3 (high)** | 138 (54·8)  75 (29·8)  37 (15·5) | 115 (44·7)  78 (30·4)  64 (24·9) | Ref  0·80 (0·54-1·20)  0·51 (0·32-0·81) | **0·005†** | Ref  1·03 (0·60-1·75)  0·68 (0·36-1·26) | 0·132† |
| **Critical care admission** | 20 (7·9) | 21 (8·2) | 0·97 (0·51-1·83) | 0·92 | 1·00 (0·66-1·55) | 0·97 |
| **30-day mortality** | 15 (6·0) | 25 (9·7) | 0·58 (0·30-1·13) | 0·11 | 0·74 (0·59-0·95) | **0·02** |
| **30-day readmission** | 26 (10·3) | 44 (17·1) | 0·51 (0·29-0·89) | **0·02** | 0·51 (0·26-0·1·03) | **0·06** |
| **Subgroup analysis for specific risk groups** | | | | | | |
| **16-64 years, no clinical risk factor** | 80 (31·7) | 47 (18·3) | 2·08 (1·38-3·14) | **0·001** | 1·54 (1·10-2·15) ^a^ | **0·01** |
| **16-64 years, ≥1 risk factor** | 49 (19·4) | 40 (15·6) | 1·31 (0·83-2·07) | 0·25 | 1·10 (0·56-2·20) ^a^ | 0·78 |
| **≥65 years, no clinical risk factor** | 26 (10·3) | 45 (17·5) | 0·54 (0·32-0·91) | **0·02** | 0·60 (0·32-1·09) ^a^ | 0·09 |
| **≥65 years, ≥1 risk factor** | 97 (38·5) | 124 (48·2) | 0·67 (0·47-0·95) | **0·03** | 0·91 (0·60-1·29) ^a^ | 0·68 |
| **Pneumococcal clinical risk group**‡ | 172 (68·2) | 209 (81·3) | 0·78 (0·55-1·16) | 0·18 | 0·98 (0·67-1·41) ^a^ | 0·90 |

STs serotypes; OR Odds ratio; aOR adjusted odds ratio; IQR Interquartile range CCF Congestive cardiac failure; COPD Chronic obstructive pulmonary disease

Data are presented as n (%). Bold values indicate p-value <0.05.

*Adjusted for age, gender, residential care, co-morbidities, ppv23 vaccine **Adjusted for age, gender, residential care, co-morbidities, ppv23 vaccine, and site

†p-value for trend

‡Pneumococcal clinical risk group includes everyone aged ≥65 years, plus anyone aged 16-64 years with one or more of the following clinical risk factors: chronic respiratory disease, chronic heart disease, chronic liver disease, chronic kidney disease, diabetes requiring treatment, immunosuppression (immunosuppressive medications, previous solid organ transplant, previous bone marrow transplant, splenic dysfunction, neoplastic disease with active treatment in last 6 months, haematological malignancy, primary immunodeficiency, HIV), CSF leak or cochlear implant)

^a^Adjusted for gender, residential care, ppv23 vaccine receipt clustered by site, number in model = 509

# Supplementary Figure 1

Supplementary Figure 1 Viral pathogens identified in patients with community-acquired pneumonia, as a percentage of those in which a viral throat swab was tested

RSV: respiratory syncytial virus

HSV: Herpes simplex virus

Mixed viral: adenovirus/parainfluenza (n=1); adenovirus/rhinovirus (n=2); influenza A/enterovirus (n=1); influenza A/metapneumovirus (n=1); influenza A/ RSV (n=1); influenza B/Metapneumovirus (n=1); parainfluenza/Metapneumovirus (n=1); rhinovirus/enterovirus (n=43); rhinovirus/enterovirus/metapneumovirus (n=1); bocavirus/rhinovirus (n=1); enterovirus/adenovirus (n=1)

Bacteria identified (%) from blood cultures performed (n=1394):

| *S pneumoniae* | 78 (5·6) |
| --- | --- |
| *Staphylococcus aureus* | 8 (0·6) |
| *Escherichia coli* | 8 (0·6) |
| *Streptococcus pyogenes* | 4 (0·3) |
| *Haemophilus influenzae* | 2 (0·14) |
| *Moraxella catarrhalis* | 2 (0·14) |
|  |  |
| Staphylococci (non *S aureus*) | 50 (3·6) |
| Non-haemolytic streptococci | 17 (1·2) |
| Mixed growth | 14 (1·0) |
| Other bacteria* | 12 (0·9) |

*Other bacteria (all n=1): Bacillus spp; *Citrobacter koseri*; Prevotella spp; *Pseudomonas aeruginosa; Serratia marcescens; Corynebacterium irritans*; diphtheroids; *Enterococcus faecalis; Klebsiella oxytoca; Mycoplasma pneumoniae*; Propionibacterium spp; *Moraxella osleosis*

# Supplementary Figure 2

*Supplementary Figure 2 Bubble plot of first and second pneumococcal serotypes/serogroups in 149 people with ≥2 serotypes identified by Bio-Plex24 assay. Area of the circle is proportional to the frequency of each serotype/serogroup combination*

# Supplementary Figure 3

Supplementary Figure 3 Serotypes detected amongst pneumococcal blood cultures as a percentage of all serotyped isolates (n=70). Results shown as percentages in graph and actual numbers in table.

| Serotype | Frequency |
| --- | --- |
| 8 | 24 |
| 3 | 10 |
| 9N | 8 |
| 19A | 6 |
| 12F | 5 |
| 22F | 4 |
| 16F | 3 |
| 7C | 1 |
| 10A | 1 |
| 11A | 1 |
| 13 | 1 |
| 15A | 1 |
| 17F | 1 |
| 23A | 1 |
| 24F | 1 |
| 33F | 1 |
| 35B | 1 |

1. Eletu SD, Sheppard CL, Rose S, Smith K, Andrews N, Lim WS, et al. Re-validation and update of an extended-specificity multiplex assay for detection of Streptococcus pneumoniae capsular serotype/serogroup-specific antigen and cell-wall polysaccharide in urine specimens. Access microbiology. 2020;2(3):acmi000094. [↑](#footnote-ref-2)
